# Annual acknowledgement of manuscript reviewers

**DOI:** 10.1186/s12995-015-0045-x

**Published:** 2015-02-07

**Authors:** Axel Fischer, David A Groneberg

**Affiliations:** Journal of Occupational Medicine and Toxicology, ᅟ, ᅟ

## Abstract

The editors of *Journal of Occupational Medicine and Toxicology* would like to thank all our reviewers who have contributed to the journal in Volume 9 (2014).

**Peter Angerer**

Germany

**Katherine Applegate**

United States of America

**Michael Bates**

United States of America

**Olof Beck**

Sweden

**Beate Beermann**

Germany

**Alessandra Binazzi**

Italy

**Elyse Bissonnette**

Canada

**Irina Boeckelmann**

Germany

**Ros Bramwell**

United Kingdom

**Lygia Therese Budnik**

Germany

**Mariana Butinof**

Argentina

**Luenda Charles**

United States of America

**Stephen Child**

New Zealand

**Emanuela Corsini**

Italy

**Virginia Cowen**

United States of America

**Reinhard Dammann**

Germany

**Sabine Darius**

Germany

**Marie de Perio**

United States of America

**M Teresa del Campo**

Spain

**Roland Diel**

Germany

**Rune Djurhuus**

Norway

**David Dorman**

United States of America

**Madeleine Dulon**

Germany

**Leslie Easterwood**

United States of America

**Bernadette Eberlein**

Germany

**Didier Ebo**

Belgium

**Peter Moeller Eggertsen**

Denmark

**Rosalie Elespuru**

United States of America

**Christina Erbe**

Germany

**Annegret Flothow**

Germany

**Simon Forstmeier**

Germany

**Priyadarshani Galappatthy**

Sri Lanka

**David A. Groneberg**

Germany

**Shamai Grossman**

United States of America

**Jim Gruber**

Canada

**Toby Hall**

Australia

**Nguyen Hang**

Viet Nam

**Noriaki Harada**

Japan

**Volker Harth**

Germany

**Jill Harvilchuck**

United States of America

**Arto Hautala**

Finland

**Paul Henneberger**

United States of America

**Mario Hermsen**

Spain

**Johan Holst**

Norway

**Ron House**

Canada

**Kirsti Husgafvel-Pursiainen**

Finland

**Ljiljana Janosevic**

Serbia

**Kate Jones**

United Kingdom

**Debra Kaden**

United States of America

**Boris Kan**

Sweden

**Naoto Keicho**

Japan

**Chandrasekharan Nair Kesavachandran**

India

**Benedikt Korf**

Switzerland

**Bartosz Koszowski**

United States of America

**Ulrich Kuch**

Germany

**Philip J. Landrigan**

United States of America

**Olivier Laurent**

France

**Gerald Li**

Canada

**Hester Lipscomb**

United States of America

**Brian Lowe**

United States of America

**Chao Lu**

United States of America

**Alwin Luttmann**

Germany

**Stephanie MacNeill**

United Kingdom

**Anne Mette Madsen**

Denmark

**Suzanne Marsh**

United States of America

**Bernard Martin**

United States of America

**Luca Mascitelli**

Italy

**Giuseppe Mastrangelo**

Italy

**Denise Matos**

Brazil

**Gaetane Michaud**

United States of America

**Marin Mladinic**

Croatia

**Bente Moen**

Norway

**Diego Moliner-Urdiales**

Spain

**Andrew Moore**

United Kingdom

**Peter Morfeld**

Germany

**Albert Nienhaus**

Germany

**Matthias Nuebling**

Germany

**Daniela Ohlendorf**

Germany

**Karin Ollinger**

Sweden

**Christian Ostheimer**

Germany

**Cheryl Peters**

Canada

**Ceri Phillips**

United Kingdom

**Lisa Pompeii**

United States of America

**Alexandra Marita Preisser**

Germany

**Heribert Ramroth**

Germany

**David Rees**

South Africa

**Sarah Rodgers**

United Kingdom

**Erwin L Roggen**

Denmark

**Chameen Samarawickrama**

Australia

**Riitta Sauni**

Finland

**Ulf Martin Schilling**

Sweden

**Annina Schmid**

United Kingdom

**Peter-Ernst Schnabel**

Germany

**Ashley Schoenfisch**

United States of America

**Peter William Schofield**

Australia

**Johannes Schulze**

Germany

**Andreas Seidler**

Germany

**G Shi**

China

**Dennis Shusterman**

United States of America

**Mareike Simon**

Germany

**Maria Fernanda Simoniello**

Argentina

**Lidwien Smit**

Netherlands

**Robert Tarone**

United States of America

**Richard Telford**

Australia

**Beatrice Thielmann**

Germany

**Francesco Tomei**

Italy

**José Torres da Costa**

Portugal

**Dominique Tripodi**

France

**Jukka Uitti**

Finland

**Victoria Vivilaki**

Greece

**Valerie Walker**

United Kingdom

**Eileen M. Wanke**

Germany

**Hanna Watling**

Australia

**Heather Webb**

United States of America

**Sabine Wicker**

Germany

**Michael Witcher**

Canada

**Rosalind Wright**

United States of America

**Athanasios Xanthopoulos**

Germany

**Il Je Yu**

Korea, South

**Alice Zwerling**

United States of America

